# Cystathionine β-Synthase Inhibition Is a Potential Therapeutic Approach to Treatment of Ischemic Injury

**DOI:** 10.1177/1759091415578711

**Published:** 2015-03-30

**Authors:** Su Jing Chan, Chou Chai, Tze Wei Lim, Mie Yamamoto, Eng H Lo, Mitchell Kim Peng Lai, Peter Tsun Hon Wong

**Affiliations:** 1 Department of Pharmacology, Yong Loo Lin School of Medicine, National University Health System, National University of Singapore, Singapore; 2 Duke-NUS Graduate Medical School, Singapore; 3 Departments of Radiology and Neurology, Massachusetts General Hospital, Harvard Medical School, Charlestown, MA, USA

**Keywords:** cystathioine β-synthase, hydrogen sulfide, homocysteine, cysteine, oxygen glucose deprivation, stroke

## Abstract

Hydrogen sulfide (H_2_S) has been reported to exacerbate stroke outcome in experimental models. Cystathionine β-synthase (CBS) has been implicated as the predominant H_2_S-producing enzyme in central nervous system. When SH-SY5Y cells were transfected to overexpress CBS, these cells were able to synthesize H_2_S when exposed to high levels of enzyme substrates but not substrate concentrations that may reflect normal physiological conditions. At the same time, these cells demonstrated exacerbated cell death when subjected to oxygen and glucose deprivation (OGD) together with high substrate concentrations, indicating that H_2_S production has a detrimental effect on cell survival. This effect could be abolished by CBS inhibition. The same effect was observed with primary astrocytes exposed to OGD and high substrates or sodium hydrosulfide. In addition, CBS was upregulated and activated by truncation in primary astrocytes subjected to OGD. When rats were subjected to permanent middle cerebral artery occlusion, CBS activation was also observed. These results imply that in acute ischemic conditions, CBS is upregulated and activated by truncation causing an increased production of H_2_S, which exacerbate the ischemic injuries. Therefore, CBS inhibition may be a viable approach to stroke treatment.

## Introduction

Hydrogen sulfide (H_2_S) has been known as a toxic gas for more than a century due to its reversible inhibition of cytochrome c oxidase (Beauchamp et al., 1984; Reiffenstein et al., 1992; Dorman et al., 2002). In recent years, it has been recognized that H_2_S functions as a signaling molecule in the central nervous system (CNS), involving in the regulation of ion channels, neurotransmitter functions, and other intracellular signaling molecules such as tyrosine kinases (Abe and Kimura, 1996; Tan et al., 2010; Liu et al., 2012). Current evidence suggests that H_2_S is predominantly produced in the CNS by cystathionine β-synthase (CBS) using cysteine (Cys) and homocysteine (Hcy) as substrates (Abe and Kimura, 1996; Chen et al., 2004; Singh et al., 2009). CBS is heme and pyridoxal-5′phosphate dependent. It is predominantly expressed in astrocytes (Enokido et al., 2005; Lee et al., 2009) but has also been reported to be expressed in Purkinje cells and Ammon's horn neurons (Robert et al., 2003).

Cys has been reported as a risk factor for coronary heart disease (Xiao et al., 2011), while Hcy is known as a risk factor for stroke (Hankey, 2006). A prospective study showed that total Hcy concentration was significantly higher in stroke cases than in controls (geometric mean 13.7 vs. 11.9 μM; Perry et al., 1995). Interestingly, high Hcy concentration in blood of ischemic stroke patients is associated with a negative influence on stroke outcome (Pniewski et al., 2003). Similarly, high plasma Cys levels on admission before treatment also correlated significantly with poor clinical outcome assessed at 3 months post stroke (Wong et al., 2006). These findings implicated a clinical relevance of Hcy and Cys in ischemic stroke outcome. In addition, Cys is a substrate for γ-glutamylcysteine synthetase, which catalyzes the first reaction in the biosynthesis of glutathione (GSH), a major cellular antioxidant known to protect cells from oxidative damage mainly through the action of GSH peroxidase (Cohen and Hochstein, 1963; Meister and Anderson, 1983). This reaction is ATP dependent and therefore GSH will be depleted following ATP depletion. This may contribute to an increase in Cys level under ischemic conditions.

In animal studies, Qu et al. (2006) reported that H_2_S was increased in the cerebral cortex at 24 hr after permanent middle cerebral artery occlusion (pMCAO), a rat stroke model. The H_2_S level was further increased by Cys administration. Pretreatment with Cys increased the infarct volume following pMCAO, while the inhibition of H_2_S synthesis by CBS inhibitors reduced infarct volume. Moreover, sodium hydrosulfide (NaHS, an H_2_S donor) could mimic the effects of Cys pretreatment. These results suggested that in ischemia, H_2_S production increases through increased availability of Cys and increased H_2_S worsens ischemic injuries, based on both histological and functional assessments (Qu et al., 2006). Therefore, suppression of H_2_S production via the inhibition of CBS activity may be an attractive approach to improve stroke outcome during the acute phase of ischemic stroke. However, inhibition studies were performed using nonselective inhibitors of CBS (Asimakopoulou et al., 2013). An often used inhibitor, aminooxyacetic acid (AOAA) also inhibits cystathioine γ-lyase (CSE) and other enzymes like γ-aminobutyric acid (GABA) transaminase (Loscher, 1981). Furthermore, the possibility of pleiotropic effects of these inhibitors in influencing stroke outcome cannot be entirely ruled out.

In order to further establish if the overproduction of endogenous H_2_S leads to enhanced cell death under ischemic conditions, we overexpressed CBS in undifferentiated neuroblastoma SH-SY5Y cells. Enhanced cell death was observed when the cells were subjected to oxygen and glucose deprivation (OGD) in the presence of Cys and Hcy. The same was then observed in primary astrocytes albeit to a lesser extent. In addition, we found that OGD rapidly increased the expression of CBS in primary astrocytes and in rat brains subjected to pMCAO. Hence, we provide evidence that upregulation of CBS expression exacerbates the outcome of stroke most likely through increased H_2_S production.

## Methods

### Ethics Statement

All animal experimental procedures in this study were approved by the Institutional Animal Care and Use Committee of the National University of Singapore.

### Cell Culture

Neuroblastoma SH-SY5Y cell line was obtained from American Type Culture Collection (ATCC). Cell were maintained in Dulbecco's modified Eagle's medium (DMEM)/F12 (Gibco, Invitrogen, USA) containing 10% fetal bovine serum (FBS; Gibco, Invitrogen, USA) and 1% penicillin–streptomycin (Gibco, Invitrogen, USA) at 37 °C and incubated in a humidified atmosphere with 95% air/5% CO_2_.

### Primary Culture of Rat Cortical Astrocyte

Primary astrocyte cultures were prepared from the cerebral cortices of 1- to 2-day old Sprague-Dawley rat pups as described previously (Lu et al., 2008) with minor modifications. Briefly, the cerebral cortices were harvested and digested with 0.25% trypsin (Gibco, Invitrogen, USA) at 37 °C. The cells were seeded onto poly-l-lysine-coated flask with DMEM/F12 (Gibco, Invitrogen, USA) medium supplemented with 10% FBS (Gibco, Invitrogen, USA) and 1% penicillin–streptomycin (Gibco, Invitrogen, USA). The cultures were maintained at 37 °C in a humidified atmosphere with 95% air/5% CO_2_. Culture medium was replaced 72 hr later and changed medium twice weekly thereafter. At 10 to 12 days, the confluent cultures were shaken overnight to minimize microglia contamination. The remaining astrocyte monolayers were cultured until Day 21.

### Construction of Rat CBS Lentiviral Expression Vectors

Total RNA was isolated from rat PC12 cells using RNeasy Mini Kit (Qiagen). First-strand cDNA was synthesized from total RNA using the RevertAid H Minus First Strand cDNA Synthesis Kit (Fermentas/Thermo Scientific). CBS coding region was isolated by polymerase chain reaction (PCR) from the cDNA using PrimeStar GXL DNA Polymerase (Clontech) with a SalI site-containing forward (5′ CGCGTCGACCATG CCTTCAGGGACATCC 3′) and BamHI site-containing reverse (GCGGATCCTATTTCCGGGTCTGCTCAC) primers. Amplified product was digested with SalI and BamHI and inserted in-frame 3′ to a myc-tag sequence in pL6mCWmycIE lentiviral vector to give pL6mCWmycCBSIE. pLenti6mCWmycIE was modified from pLenti6/V5-D-TOPO (Invitrogen) by reengineering the multiple cloning site, insertion of the cPPT and WPRE elements, and insertion of the N-terminal myc tag coding sequence and IRES-EGFP reporter cassette.

### Lentivirus Particle Production and Cell Transduction

Lentivirus packaging was performed in 293FT cells according to the protocol provided with the ViraPower™ Lentiviral Directional TOPO® Expression Kit (Invitrogen). Lentivirus particles were harvested from cell culture supernatant according to the protocol of Deiseroth Lab (http://www.stanford.edu/group/dlab/resources/lvprotocol.pdf). Lentivirus harboring the CBS expression constructs was used to transduce undifferentiated SH-SY5Y cells. Prior to transduction, cells were cultured to 90% confluence. Concentrated virus particle was added to cell culture medium containing 8 μg/ml of Polybrene (Sigma Aldrich). Where long-term expression of transgene was needed, antibiotic selection was applied by adding Blasticidin S (Invitrogen) at a final concentration of 10 μg/ml to the medium. Expression of transgene was visualized by EGFP fluorescence.

### Oxygen and Glucose Deprivation

OGD was achieved by incubating the SH-SY5Y cells with and without CBS overexpression in glucose-free DMEM with 1% streptomycin/penicillin in a hypoxia chamber (1% O_2_/5% CO_2_/94% N_2_) for 24 hr at 37 °C in a humidified incubator. OGD for primary cortical astrocyte culture was achieved by incubating the primary astrocytes in glucose-free DMEM with 1% streptomycin/penicillin in a hypoxia chamber (5% CO_2_/95% N_2_) from 0.5 to 8 hr at 37 °C in a humidified incubator. For NaHS treatment, primary astrocytes were seeded onto 96-well plate overnight. The regular medium was replaced with OGD medium supplemented with vehicle or NaHS at 100 or 300 µM. Cell viability was measured using the 3-(4,5-dimethylthiazol-2-yl)-2,5-diphenyltetrazolium bromide (MTT) assay (0.5mg/ml, 2.5 hr) with formazan solubilized in 100% dimethylsulfoxide (DMSO) and absorbance determined at 570 nm using a microplate reader (Sunrise; TECAN); and by lactate dehydrogenase (LDH) assay using a Cytotoxicity Detection Kit (Roche) according to the protocol recommended by the manufacturer. Total LDH release was obtained by treating control cells with 2% Triton X-100 (Sigma). Absorbance at 490 nm was determined after 30-min incubation. Percentage of LDH release was measured as [(experimental value − vehicle control)/ (total release − vehicle control)] × 100%.

### H_2_S Synthesizing Activity Assay

H_2_S synthesizing activity in cells was measured according to Qu et al. (2006). Briefly, cells were homogenized in 0.1 mol/L potassium phosphate buffer, pH 7.4 (0.45 ml) containing varying concentrations of Cys and Hcy (total volume 0.5 ml). Blank was done by omitting the substrates. After incubation for 90 min at 37 °C, zinc acetate (1%, 0.25 ml) was added into the reaction mixture followed by tricholoracetic acid (10%, 0.25 ml). After centrifugation (13,000 rpm, 10 min) at 4 °C, N-dimethyl-p-phenylenediamine sulfate (20 mmol/L prepared in 7.2 mol/L HCl, 0.133 mL) and FeCl_3_ (30 mmol/L prepared in 1.2 mol/L HCl, 0.133 mL) were added to the supernatant. Absorbance at 670 nm was measured using a microplate reader (Sunrise; TECAN) 20 min later. Absorbance was converted to H_2_S concentration through a standard curve obtained by using NaHS as the standard.

### pMCAO Model

pMCAO was performed according to Tamura et al. (1981). Animals were housed under diurnal lighting conditions and fed standard rat chow and water ad libitum. Cerebral ischemia was induced by permanent occlusion of the left middle cerebral artery (MCA) using a subtemporal approach. Male Sprague-Dawley rats (6 to 8 weeks old) were anesthetized with ketamine (75 mg/kg, intraperitoneally; Parnell Laboratories Pty Ltd, Alexandria, NSW, Australia) and xylazine (10 mg/kg, intraperitoneally; Troy Laboratories Pty Ltd, Smithfield, NSW, Australia). The MCA was exposed through a subtemporal craniectomy and cauterized from the point proximal to its origin to the points where it intersects the inferior cerebral vein. During the procedure, rat body temperature was maintained at 37 °C until recovery from anesthesia. For infarct volume assessments, rats were decapitated at 8 or 24 hr after the pMCAO procedure as described above. Whole brains were cut into 2 mm coronal sections and immediately stained with 2% 2,3,5-triphenyltetrazolium chloride (TTC; Sigma Chemical Co.). Infarct volumes in the cortex, striatum, and whole hemisphere were measured using the Scion imaging software (Frederick, MD, USA), and correction for brain edema was performed using the contralateral hemisphere as control. Infarct volumes are presented as the percentage of the total volume of the brain region/hemisphere.

### Western Blot

Brains tissues from sham-operated control and pMCAO (3 to72 hr) rats were lysed by RIPA buffer (Cell Signaling) supplemented with protease inhibitor and phosphatase inhibitor cocktail (Roche, Mannheim, Germany). SH-SY5Y cells and primary cortical astrocytes were lysed by CST buffer (Cell Signaling) supplemented with protease inhibitor and phosphatase inhibitor cocktail (Roche, Manheim, Germany). Total protein was determined by Bradford protein assay (Biorad, CA, USA). Proteins were separated by 10% SDS/PAGE, transferred onto a polyvinylidene difluoride (PVDF) membrane (Amersham Biosciences, Buckinghamshire, UK), and then blocked with 10% nonfat milk. The membrane was then incubated with antibodies against CBS, or β-actin (Cell Signaling Technologies, Beverly, MA, USA) at 4 °C overnight, then washed and incubated in HRP-conjugated anti-rabbit or mouse IgG at room temperature for 1 hr. Visualization was carried out using Luminata Forte or Crescendo western HRP substrate (Millipore Corporation, Billercia, MA, USA), and the chemiluminescence signals were detected using UVIchemi (UVItec, Cambridge, UK).

### Immunofluorescence for Brain Slices

Rats were anaesthetized and perfused with 0.1 mol/L phosphate buffer saline followed by 4% paraformaldehyde through the heart. Brains were removed and immersed in 4% paraformaldehyde overnight at 4 °C. Paraformaldehyde-fixed brains were then cryoprotected in 10% sucrose and eventually 20% sucrose at 4 °C until use. Coronal sections (30 μm) were made using a cryostat and mounted on glass slides (Matsunami, Japan). Nonspecific binding was blocked by incubating the section in 5% goat serum for 1 hr at room temperature. Antibodies against CBS (1:200) was used and incubated at 4 °C overnight. Sections were then rinsed and incubated with Alexa 488-conjugated goat anti-rabbit (Molecular probes, USA) secondary antibody for 1 hr at room temperature. Fluorescent-labeled sections were mounted using ProLong gold antifade reagent (Life technologies, USA). Negative control was performed by probing sections with PBS with 0.1% Triton-X overnight. All the fluorescent images were captured using an Olympus FluoView FV1000 (Olympus, Japan) laser scanning confocal microscope, and images captured was processed by FV10-ASW1.7.

### Immunocytochemistry for Primary Cortical Astrocytes

Primary astrocyte culture on poly-l-lysine-coated glass coverslips were rinsed twice with PBS, pH 7.4, and fixed with 4% paraformaldehyde for 10 min. Fixed cells were made permeable by incubating in PBS containing 0.1% Triton-X for 10 min. Cells were blocked with 10% goat serum for 1 hr at room temperature and then incubated with primary monoclonal anti-GFAP (1:400; Sigma Aldrich, USA) antibody and anti-CBS (1: 200) antibody at 4 °C overnight. After washing with PSB, cells were incubated with Alexa 488-conjugated goat anti-rabbit (Molecular probes, USA) or Alexa 555 (Molecular probes, USA) for 1 hr at room temperature, and coverslip was mounted using ProLong gold antifade reagent (Life technologies, USA). Fluorescent images were captured using QImaging (Canada), and images captured were processed by Image Pro insight (QImaging, Canada).

### Statistical Analysis

All comparisons were performed by one-way analysis of variance (ANOVA) followed by post hoc analysis with Bonferroni correction or by two-tailed independent *t* test using IBM SPSS Statistics 19. Data are expressed as mean±*SEM*. Statistical significance is reached when *p* < .05.

## Results

### CBS Overexpression in SH-SY5Y Cells

**Figure 1.**
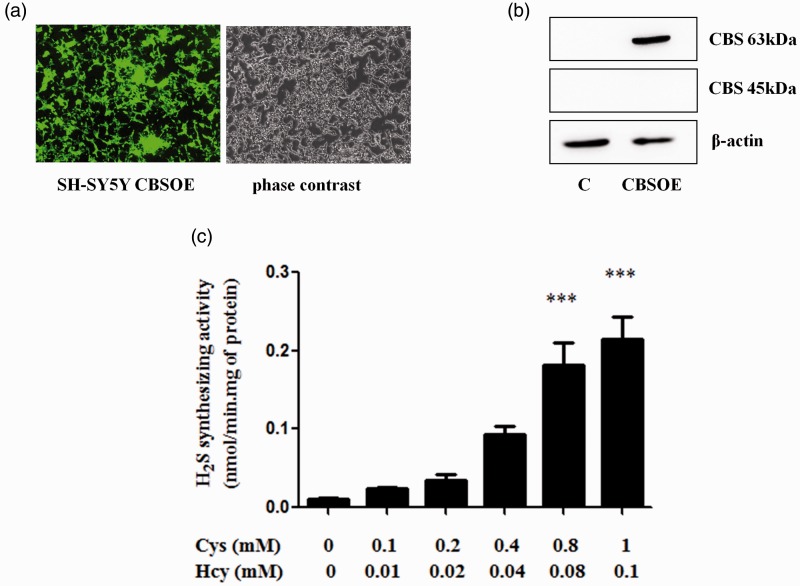
Lentiviral vector transduced CBS-overexpressing (CBSOE) SH-SY5Y cells. (a) Fluorescence (left) and phase contrast (right) micrographs of SH-SY5Y CBSOE cells. Fluorescence indicates CBS-EGFP expression. (b) Western blot results confirming that CBS was markedly expressed compared with nontransduced control (C) cells. Only the full-length CBS (63 kDa) was detected but not the truncated CBS (45 kDa). (c) H_2_S synthesizing activity of SH-SY5Y CBSOE cells measured at varying concentrations of cysteine (Cys) and homocysteine (Hcy). Data are presented as mean ± *SEM, n* = 3. ANOVA: *F*(5, 12) = 25.125, *p* < .05; ***p < .001 against no substrate control by Bonferroni. CBS = cystathionine β-synthase; H_2_S = hydrogen sulfide; ANOVA = analysis of variance; CBSOE = CBS-overexpressing; Cys = cysteine; Hcy = homocysteine.

Under normal conditions, SH-SY5Y cells express CBS at a very low level. However, they can be efficiently transduced with lentiviral vector to overexpress CBS together with EGFP, a fluorescent reporter. Results shown in Figure 1 confirmed that these transduced cells strongly express both EGFP and CBS (Figure 1(a) and 1(b)). The full-length 63 kDa isoform was detected by Western blot but not the truncated 45-kDa isoform (Figure 1(b)), indicating that the truncation mechanism may not be in place in these cells. When CBS substrates (Cys + Hcy) were added to cell homogenates, H_2_S was produced in a concentration-dependent manner (Figure 1(c)). These results indicate that the CBS artificially overexpressed in these cells are fully functional. As 0.1 mM Cys + 0.01 mM Hcy are reportedly close to physiological concentrations (Singh et al., 2009; Stipanuk and Ueki, 2011), these concentrations were used as the low substrate condition and a 10-fold increase as the high substrate condition in subsequent experiments.

### Effects of H_2_S Production on Cell Viability

**Figure 2.**
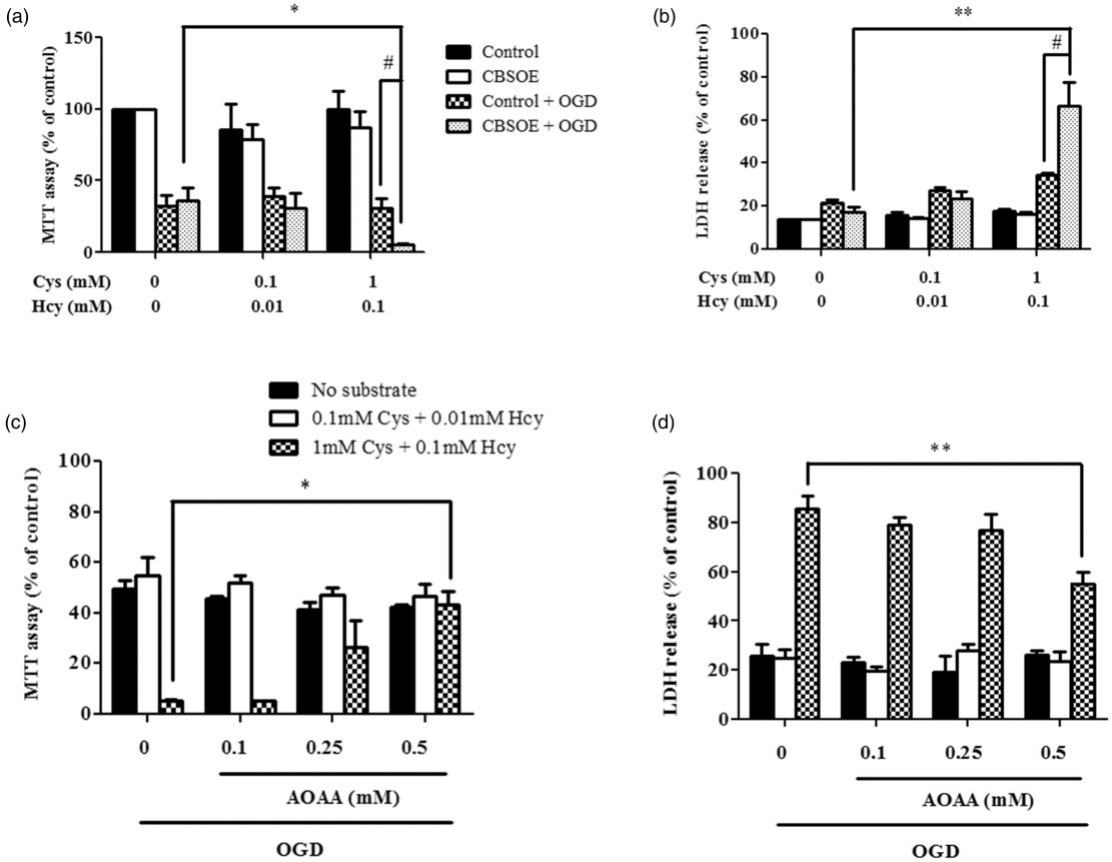
The effects of OGD on cell viability of CBSOE cells under various conditions that affected H_2_S production. (a and b) MTT assay and LDH release results are used as indicators of cell viability. CBSOE or control SH-SY5Y cells were exposed to either no, low (0.1 mM Cys + 0.01 mM Hcy), or high (1 mM Cys + 0.1 mM Hcy) substrate concentrations with or without 24 hr OGD. Control cells without OGD (a) or cells treated with 2% Triton (b) were used as 100%. ANOVA for CBSOE cells: *F*(2, 9) = 6.327, *p* < .05 (a) and *F*(2, 6) = 15.000, *p* < .01 (b). *p < .05, **p < .001 against CBSOE cells subjected to OGD without substrates by Bonferroni; #*p* < .05 against control cells subjected to OGD in the presence of high substrates by independent *t* test. Data are mean ± *SEM, n* = 3–4. (c and d) CBS inhibition by AOAA reversed the enhanced cell death in CBSOE cells subjected to OGD in the presence of high substrates. Cell viability is expressed as fraction to control without OGD. ANOVA for high substrate conditions: *F*(3, 8) = 9.799 (c) and *F*(3, 12) = 7.322 (d), *p* < .01. *p < .05, **p < .001 against without AOAA by Bonferroni. Data are mean ± *SEM, n* = 3–4. OGD = oxygen and glucose deprivation; CBSOE = CBS-overexpressing; H_2_S = hydrogen sulfide; MTT = 3-(4,5-dimethylthiazol-2-yl)-2,5-diphenyltetrazolium bromide; LDH = lactate dehydrogenase; ANOVA = analysis of variance; CBS = cystathionine β-synthase; AOAA = aminooxyacetic acid; Cys = cysteine; Hcy = homocysteine.

CBS-overexpressing (CBSOE) cells did not show any difference in cell viability on exposure to high or low substrate concentrations when compared with control cells as shown by the MTT assay (Figure 2(a)). However, when cells are subjected to OGD, cell death increased markedly regardless of substrate conditions. In the absence or presence of low substrates, cell viability fell to about 30% to 40 % in both cell types whether or not CBS was overexpressed. In the presence of high substrates, cell viability was about 30% in the control cells but about 5% in the CBSOE cells, indicating a significant increase in cell death. When LDH release was measured under the same conditions, the results were entirely consistent with the MTT assay results (Figure 2(b)).

The enhanced cell death observed in CBSOE cells when exposed to high substrate condition was attenuated concentration in a concentration-dependent manner by the addition of AOAA, a nonselective CBS inhibitor, immediately after the addition of substrates (Figures 2(c) and (d)). At 0.5 mM of AOAA, complete reversal was achieved in the MTT assay, while only partial reversal was observed in LDH release assay. These data strongly support the notion that elevated production of H_2_S in cells subjected to high substrate conditions enhanced cell death when the cells were subjected to OGD.

**Figure 3.**
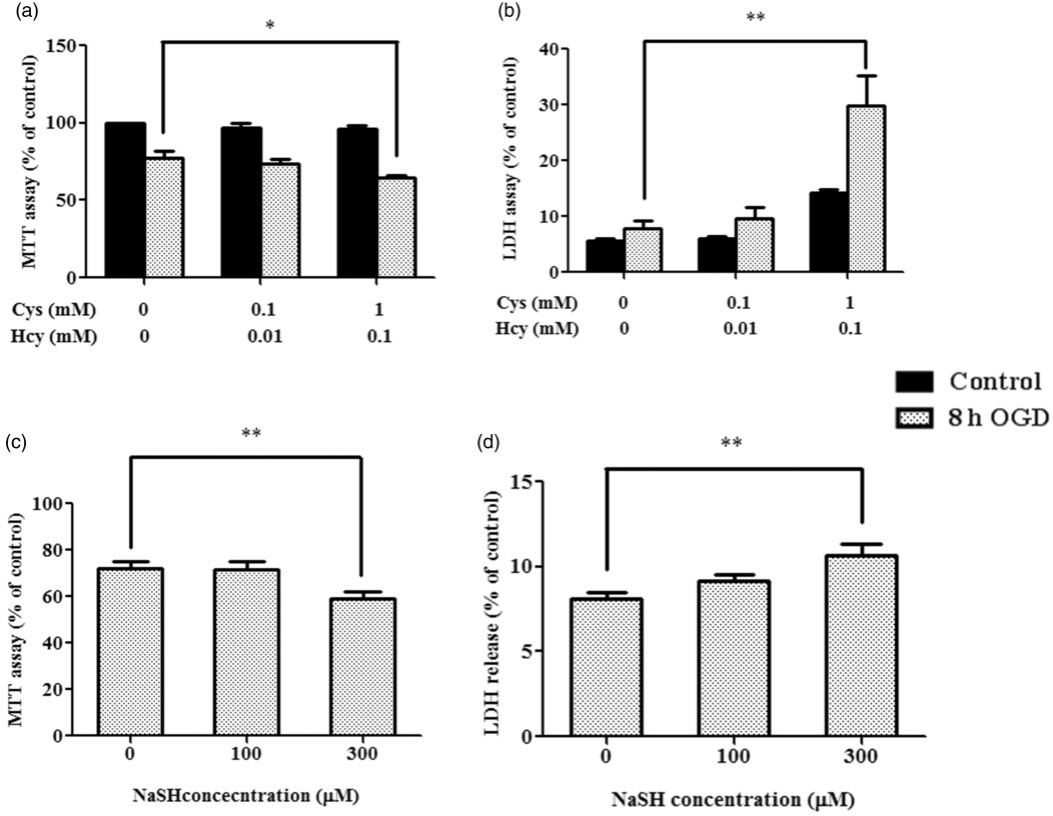
Comparison of endogenously produced and exogenous H_2_S. (a and b) MTT test and LDH release results are used as indicators of cell viability. Primary cortical astrocyte were exposed to either no, low (0.1 mM Cys + 0.01 mM Hcy), or high (1 mM Cys + 0.1 mM Hcy) substrate concentrations with or without 8 hr OGD. Control cells without OGD (a) or cells treated with 2% Triton (b) were used as 100%. Data are presented as mean ± *SEM, n* = 3–4. ANOVA for 8 hr OGD: *F*(2, 9) = 5.013, *p* < .05 (a) and *F*(2, 9) = 12.107, *p* < .01 (b). *p < .05, **p < .01 against no substrates by Bonferroni. (c and d) Primary cortical astrocytes were exposed to either 100 or 300 µM NaHS and then subjected to 8 hr OGD. Data are presented as mean ± *SEM, n* = 4–6 (c) or 8 (d). ANOVA: *F*(2, 12) = 8.065 (c) and *F*(2, 21) = 6.988 (d), *p* < .01. **p < .01 against without NaHS by Bonferroni. H_2_S = hydrogen sulfide; MTT = 3-(4,5-dimethylthiazol-2-yl)-2,5-diphenyltetrazolium bromide; LDH = lactate dehydrogenase; OGD = oxygen and glucose deprivation; ANOVA = analysis of variance; Cys = cysteine; Hcy = homocysteine.

We next examined the effects of OGD in primary rat astrocytes under different substrate conditions. Consistent with the findings in CBSOE SH-SY5Y cells, primary astrocytes subjected to OGD under high concentrations of Cys + Hcy showed cell viability of about 64% by MTT assay, a significant decrease from about 77% in cells not exposed to substrates (Figure 3(a)). This is supported by a threefold increase in LDH release (Figure 3(b)). A small but significant increase in LDH release was also observed in control cells exposed to the high substrate condition without OGD, as opposed to no effects observed by MTT assay. This may be due to toxicity attributable to Cys directly (Janaky et al., 2000) or indirectly (Nath and Salahudeen, 1993). To see if these effects could be mediated by H_2_S, we added NaHS at 300 mM and observed a significant decrease in cell viability in primary astrocytes when subjected to OGD (Figures 3(c) and (d)).

### Upregulation of CBS Expression and Infarct Volume Following Ischemic Insults

**Figure 4.**
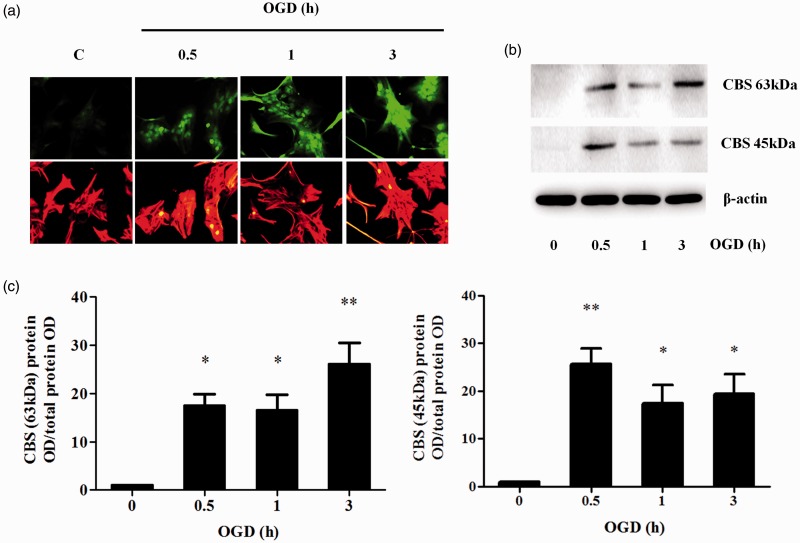
Effects of OGD on CBS expression in primary cultures of cortical astrocytes. (a) Representative immunocytochemical staining of primary cortical astrocytes after OGD (0.5 to 3 hr) for CBS (green) and GFAP (red). (b) Representative Western blot images showing the increased expression of both the full length CBS (63 kDa) and truncated CBS (45 kDa) after OGD. (c) Densitometry measurement of full length (63 kDa) CBS and truncated (45 kDa) CBS expression after OGD. Data are presented as mean ± *SEM, n* = 3. ANOVA for CBS 63 kDa: *F*(3, 8) = 12.193, *p* < .01. *p < .05; **p < .01 against without OGD by Bonferroni. ANOVA for CBS 45 kDa: *F*(3, 8) = 10.808, *p* < .05; *p < .05; **p < .01 against without OGD by Bonferroni. OGD = oxygen and glucose deprivation; CBS = cystathionine β-synthase; ANOVA = analysis of variance.

CBS expression in primary astrocytes was found to increase rapidly in rat brains within 30 min after the onset of OGD and remained high for at least 3 hr (Figure 4). However, unlike CBSOE SY-SH5Y cells, Western blot results demonstrated the presence of both the full-length and truncated isoforms (63 and 45 kDa, respectively) of CBS.

**Figure 5.**
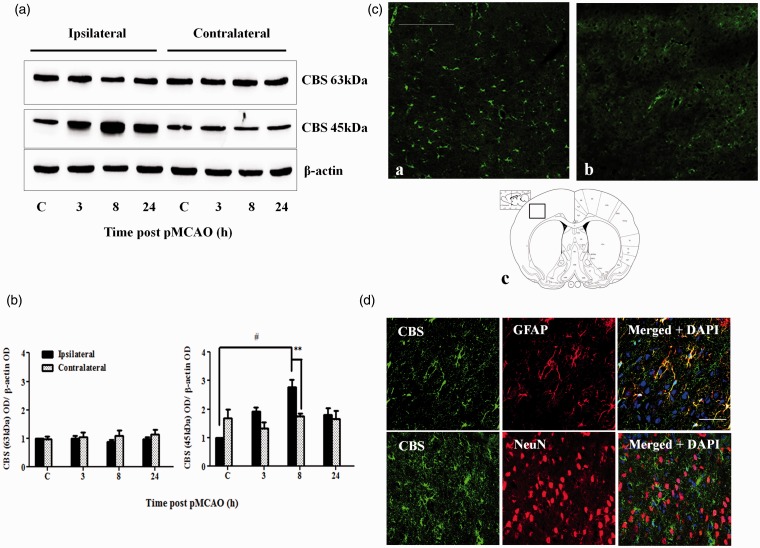
CBS expression in the cerebral cortex after pMCAO. (a) Representative Western blot results on CBS expression at 3 to 24 hr post-pMCAO in the cerebral cortex. (b) Densitometry measurement of CBS expression over 24 hr after pMCAO. Protein expression is expressed relative to the ipsilateral control C. Data are presented as mean ± *SEM, n* = 3–4. ANOVA for CBS 63 kDa on the ipsilateral side: *F*(3, 12) = 0.608, *p* = .623. ANOVA for CBS 45 kDa on the ipsilateral side: *F*(3, 8) = 6.702, *p* < .05; #*p* < .05 against ipsilateral control by Bonferroni; **p < .005 against the contralateral side by independent *t* test. (c) Immunofluorescent staining of CBS showed increased CBS expression in the cortex 8 hr after pMCAO (a) compared with sham-control rats (b). Scale bar: 200 µm. (c) shows the location where the CBS immunofluorescent photomicrographs were taken. (d) Colocalization of CBS (green) and GFAP (red, top panel) and lack of colocalization of CBS (green) and NeuN (red, bottom panel) in the cortex at 8 hr after pMCAO. Scale bar: 50 µm. CBS = cystathionine β-synthase; pMCAO = permanent middle cerebral artery occlusion; ANOVA = analysis of variance.

**Figure 6.**
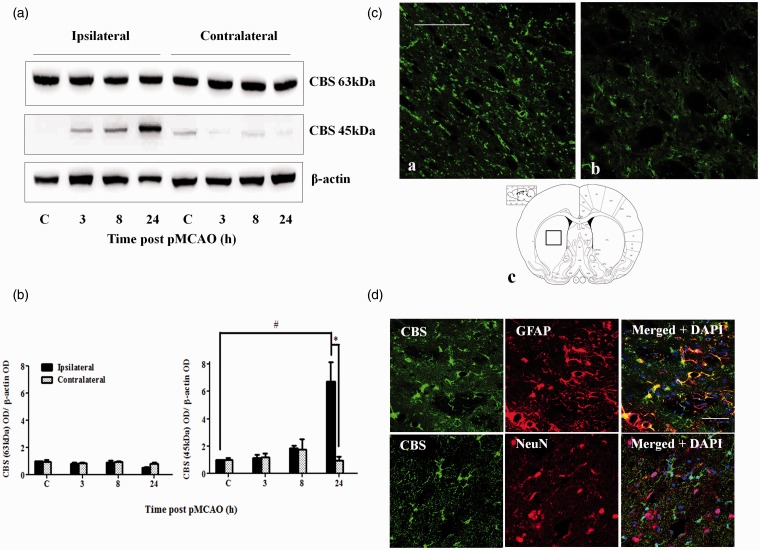
CBS expression in the striatum after pMCAO. (a) Representative Western blot results on CBS expression at 3 to 24 hr post-pMCAO in the striatum. (b) Densitometry measurement of CBS expression over 24 hr after pMCAO. Protein expression is expressed relative to the ipsilateral control C. Data are presented as mean ± *SEM, n* = 3–4. ANOVA for CBS 63 kDa: *F*(3, 8) = 3.524, *p* = .068. ANOVA for CBS 45 kDa: *F*(3, 8) = 13.637, *p* < .05; #*p* < .05 against the ipsilateral control by Bonferroni; *p < .05 against the contralateral by independent *t* test. (c) Immunofluorescent staining of CBS showed increased CBS expression in the striatum 24 hr after pMCAO (a) compared with sham-control rats (b). Scale bar: 200 µm. (c) shows the location where the CBS immunofluorescent photomicrographs were taken. (d) Colocalization of CBS (green) and GFAP (red, top panel) and lack of colocalization of CBS (green) and NeuN (red, bottom panel) in the striatum at 24 hr after pMCAO. Scale bar: 50 µm. CBS = cystathionine β-synthase; pMCAO = permanent middle cerebral artery occlusion; ANOVA = analysis of variance.

The cortex and striatum are the most affected regions following pMCAO in rats (Liu et al., 2009). Western blot analysis revealed that the truncated but not the full-length CBS was markedly upregulated (about twofold) in the cortex at 8 hr after pMCAO (Figures 5(a) and (b)). Similarly, truncated CBS expression was upregulated (sevenfold) in the striatum but at a later time point of 24 hr post-pMCAO (Figures 6(a) and (b)). Immunofluorescent staining (which does not distinguish the full length from the truncated form) results were consistent with the Western blot data, showing an upregulation of total CBS expression (Figures 5(c) and 6(c)). CBS in both regions was largely expressed in astrocytes based on colocalization with GFAP staining, while colocalization of CBS and NeuN was not observed (Figures 5(d) and 6(d)).

**Figure 7.**
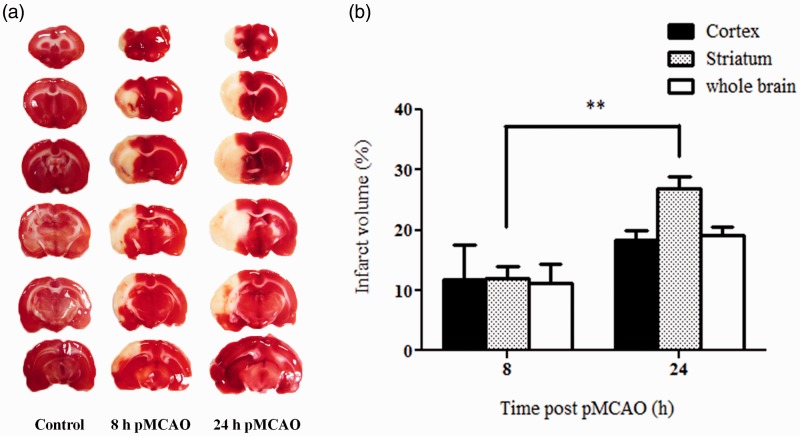
Infarct volumes at 8 and 24 hr post-pMCAO. (a) Representative TTC-stained sections showing unstained infarct areas. (b) Infarct volumes are calculated as % of total volume of the brain region after correction for edema using the contralateral hemisphere as control and presented as mean ± *SEM, n* = 3. *p < .01 by independent *t* test. TTC = 2,3,5-triphenyltetrazolium chloride; pMCAO = permanent middle cerebral artery occlusion.

In view of the different time course of CBS expression following pMCAO in the cortex and striatum, the infarct volumes in these two brain regions were determined at 8 and 24 hr post-pMCAO. It was observed that the infarct volume in the striatum was significantly larger at 24 hr than that at 8 hr (27% vs. 12%). In contrast, the infarct volume in the cortex as well as the whole affected hemisphere was not significantly different between the two time points (Figure 7). This shows that the ischemic injuries continued to develop significantly between 8 and 24 hr post-pMCAO in the striatum but not in the cortex. Therefore, the peaking of CBS expression in the striatum at 24 hr may appear to be associated with the extent of ischemic injury.

## Discussion

H_2_S can be produced in the body by three key enzymes, namely CBS, CSE, and 3-mercaptopyruvate sulfurtransferase (3-MST, in combination with cysteine aminotransferase; Kashfi and Olson, 2013). CSE is expressed predominantly in the cardiovascular system (Zhao et al., 2001; Tang et al., 2013) but at low levels in the brain and therefore generally accepted as a minor player, if at all, in H_2_S production in the brain (Abe and Kimura, 1996). 3-MST is widely present in the brain, but its physiological relevance is not clearly understood. However, in a recent study, Zhao et al. (2013) reported that 3-MST was downregulated in both the cortex and striatum following pMCAO, hence mitigating its role in increased production of H_2_S under ischemic conditions (Zhao et al., 2013). Therefore, the focus is on CBS as the predominant enzyme responsible for H_2_S production in the brain.

The native CBS enzyme is a tetramer consisting of four subunits of 63 kDa (551 amino acids). This subunit can be cleaved in its C-terminal region at Arg413 to yield a 45-kDa subunit that lacks the regulatory domain. Two truncated subunits form a dimer that is about twice as active as the tetramer (Kery et al., 1998; Majtan et al., 2014). In primary astrocytes subjected to OGD, the expression of the 63-kDa CBS was clearly upregulated (Figure 4(b)), and the truncated form increased within 30 min. Similarly, the truncated form also increased in both the cortex and striatum within 24 hr post-pMCAO (Figures 5 and 6). These observations demonstrated that CBS responds very rapidly to ischemic insults. In contrast to primary astrocytes, the full-length 63-kDa CBS in the cortex and striatum remained unchanged following pMCAO as shown by Western blotting (Figures 5(a) and 6(a)). This difference may be caused by the high basal expression in the cortex and striatum as opposed to very low basal level of CBS expression in the primary astrocytes. However, the increased level of the 45-kDa CBS implies an upregulation of the expression of the 63-kDa CBS. This is supported by the observed increase in immunostaining for CBS (Figures 5(c) and 6(c)). Therefore, it appears that increased CBS expression and activation may underlie the increased production of H_2_S in ischemia (Qu et al., 2006). The mechanism that triggers the activation by cleavage remains to be elucidated. However, it has been reported that tumor necrosis factor-α (TNF-α) induced CBS cleavage in HepG2 cells (Zou and Banerjee, 2003). This could be examined further as inflammatory cytokines such as TNF-α were found to be increased under ischemic conditions (Zhou et al., 2013). It is possible that increased CBS truncation causes a corresponding increase in the expression of the full-length enzyme by a positive feedback mechanism. It is noted that the truncated form was not detected in the SH-SY5Y CBSOE cells, indicating that cleavage of CBS does not occur or occurs only at very low level (Figure 1(b)) in cells that do not endogeneously express CBS, perhaps due to the lack of the truncation mechanism. These cells can serve as a model to investigate the factors that induce cleavage and thus activation of CBS.

Hyperhomocysteinemia is an independent risk factor for stroke (Perry et al., 1995). In addition, Hcy is elevated after stroke, and raised Hcy levels adversely affect cerebral infarction after stroke (Spence, 2007). In animal studies, Cys has been reported to increase in animal ischemic model, and Cys administration also increases the ischemic damage after stroke (Slivka and Cohen, 1993; Wong et al., 2006). Thus, there is ample evidence to suggest that in acute stroke, CBS activation is accompanied by a high availability of substrates for H_2_S production, which is most efficiently produced by β-replacement reaction where Cys condenses with Hcy to produce H_2_S (Chen et al., 2004; Singh et al., 2009). Taken together, it may be concluded that the acute ischemic conditions are conducive for markedly elevated production of H_2_S. We mimicked such conditions by overexpressing CBS in SH-SY5Y neuroblastoma cells and observed a massive increase in H_2_S production when the overexpressing cells were exposed to high substrate conditions but not substrate concentrations that may reflect normal physiological conditions (Singh et al., 2009; Stipanuk and Ueki, 2011). While Hcy and Cys can be neurotoxic to cells at high concentrations (Kruman et al., 2000; Poddar et al., 2001; Jara-Prado et al., 2003; Wong et al., 2006; Hosoi et al., 2010; Koz et al., 2010), the *high* concentrations used in these experiments were clearly nontoxic as no effects on cell viability were observed in both control or CBSOE cells. In addition, we had similarly overexpressed CBS in HEK293 cells, which are originated from the kidney. The same results were obtained in these cells as in the CBSOE SH-SY5Y cells (data not presented), indicating that the presence of a high concentration of H_2_S would enhance cell death under ischemic conditions in cells of neural or nonneural origin.

The mechanism by which H_2_S enhances cell death under ischemic conditions requires thorough investigations. It is known to inhibit cytochrome c oxidase, carbonic anhydrase, monoamine oxidase, cholinesterase, and Na^+^/K^+^-ATPase (Szabo, 2007); as well as to potentiate glutamate excitotoxicity (Cheung et al., 2007; Chen et al., 2011). Using mature mouse cortical neurons expressing functional glutamate receptors, Cheung et al. (2007) reported that glutamate-induced cell death was exacerbated by the addition of NaHS. They further reported that NaHS at concentrations < 200 µM induced apoptosis, while at concentrations > 200 µM, necrosis was induced. In contrast, Kimura and Kimura (2004) reported that H_2_S at 100 µM concentration reversed cell death in immature mouse cortical neuron treated with 1 mM glutamate (Kimura and Kimura, 2004). These findings suggest that H_2_S may have neuroprotective effects at lower range of concentrations but become cytotoxic at a higher range of concentrations (Dorman et al., 2002). In our experiments, we did not observe any protective effects under the *low* substrate conditions (Figures 2 and 3). This may be due to the low concentrations of H_2_S that we used in our experiments or the selectivity of H_2_S protective effect for excitotoxic but not ischemic insults.

*In vivo*, under ischemic conditions, excitotoxicity is more important because the failure of the energy-dependent glutamate reuptake process leads to extracellular accumulation of glutamate. However, such glutamate accumulation is more unlikely *in vitro*, so excitotoxicity is likely less important (Wang et al., 2002). OGD causes energy depletion in cells, thus interrupting ATP-dependent processes notably the Na^+^/K^+^-ATPase and eventually disruption of ion gradients across the cellular membrane. This causes cell membrane depolarization leading to Ca^2+^ overload (Wang et al., 2002) and cell death by necrosis and apoptosis (Orrenius et al., 1994; Kristian et al., 1998), involving the generation of free radicals (Abramov et al., 2007), mitochondrial or endoplasmic reticulum dysfunction (Kristian et al., 1998,) and caspase activation (Chong et al., 2012). Interestingly, the truncated CBS has also been reported to be able to generate superoxide radicals as it has reduction potential similar to other hemethiolate proteins such as chloroperoxidase, cytochrome P450, and nitric oxide synthase (Carballal et al., 2008). We are currently investigating how H_2_S may interact with the cell death mechanisms following OGD using the CBSOE cells.

H_2_S has also been reported to be cytoprotective (Calvert et al., 2010) under various other conditions. First, NaHS was reported to protect primary cortical neurons (Kimura and Kimura, 2004), primary microglia (Hu et al., 2007), and primary astrocytes (Lu et al., 2008). In these studies, glutamate, lipopolysaccharide, and hydrogen peroxide (H_2_O_2_), respectively, were used to induce cell injuries. In cultured cell lines, NaHS is reportedly protective against cell damage induced by Aβ (Tang et al., 2008), rotenone (Hu et al., 2009), 6-hydroxydopamine (6-OHDA; Tiong et al., 2010; Xie et al., 2012), and 1-methyl-4-phenylpyridinium (MPP^+^; Yin et al., 2009). In the *in vivo* setting, protective effects has been reported against global cerebral ischemia (Ren et al., 2010), and transient MCAO (Wang et al., 2014). These may indicate differences between ischemic models with and without reperfusion. Overall, it appears that the effects of H_2_S in ischemia may vary according to prevailing conditions, mechanism of the injury-inducing agent, and concentrations of H_2_S. To our knowledge, no protective effects have been reported against OGD in *in vitro* studies. Much work is needed to provide further understanding.

While it has been reported previously that administration of NaHS worsened stroke outcome in animal studies (Qu et al., 2006), we have further provided strong evidence that endogenously produced H_2_S could enhance cell death under ischemic conditions. Therefore, the present results may support the idea that CBS is a viable therapeutic target, and CBS inhibition may hold promise as a treatment of ischemic stroke. However, currently available CBS inhibitors lack selectivity and are therefore not suitable for *in vivo* investigations. More selective CBS inhibitors will be needed for further progress.

## Summary

Cystathionine β-synthase (CBS) is the predominant enzyme responsible for the increased hydrogen sulfide (H_2_S) production under ischemic conditions. High H_2_S levels leads to enhanced cell death both *in vitro* and *in vivo*. CBS may be a potential therapeutic target for the treatment of stroke.
